# Sacrificial Synthesis of Supported Ru Single Atoms and Clusters on N‐doped Carbon Derived from Covalent Triazine Frameworks: A Charge Modulation Approach

**DOI:** 10.1002/advs.202001493

**Published:** 2020-12-20

**Authors:** Zihao Zhang, Siyu Yao, Xiaobing Hu, Francis Okejiri, Kun He, Pingying Liu, Ziqi Tian, Vinayak P. Dravid, Jie Fu, Xiang Zhu, Sheng Dai

**Affiliations:** ^1^ Key Laboratory of Biomass Chemical Engineering of Ministry of Education, College of Chemical and Biological Engineering Zhejiang University Hangzhou 310027 China; ^2^ Chemical Sciences Division Oak Ridge National Laboratory Oak Ridge TN 37831 USA; ^3^ Department of Chemistry The University of Tennessee Knoxville TN 37996 USA; ^4^ Department of Materials Science and Engineering Northwestern University Evanston IL 60208 USA; ^5^ The NU*ANCE* Center Northwestern University Evanston IL 60208 USA; ^6^ Ningbo Institute of Materials Technology and Engineering Chinese Academy of Sciences Ningbo Zhejiang 315201 China; ^7^ Institute of Zhejiang University – Quzhou 78 Jiuhua Boulevard North Quzhou 324000 China; ^8^ State Key Laboratory for Oxo Synthesis and Selective Oxidation, Suzhou Research Institute of Lanzhou Institute of Chemical Physics Chinese Academy of Sciences Lanzhou 730000 China

**Keywords:** charge modulation, covalent triazine frameworks, N‐doped carbon, sintering‐resistance

## Abstract

High‐temperature pyrolysis of nitrogen (N)‐rich, crystalline porous organic architectures in the presence of a metal precursor is an important chemical process in heterogeneous catalysis for the fabrication of highly porous N‐carbon‐supported metal catalysts. Herein, covalent triazine framework (CTF) and CTF‐I (that is, CTF after charge modulation with iodomethane) are presented as sacrificial templates, for the synthesis of carbon‐supported Ru catalysts—Ru‐CTF‐900 and Ru‐CTF‐I‐900 respectively, following high‐temperature pyrolysis at 900 °C under N_2_ atmosphere. Predictably, the dispersed Ru on pristine CTF carrier suffered severe sintering of the Ru nanoparticles (NPs) during heat treatment at 900 °C. However, the Ru‐CTF‐I‐900 catalyst is composed of ultra‐small Ru NPs and abundant Ru single atoms which may have resulted from much stronger Ru—N interactions. Through modification of the micro‐environment within the CTF architecture, Ru precursor interacted on charged‐modulated CTF framework shows electrostatic repulsion and steric hindrance, thus contributing toward the high density of single Ru atoms and even smaller Ru NPs after pyrolysis. A Ru—Ru coordination number of only 1.3 is observed in the novel Ru‐CTF‐I‐900 catalyst, which exhibits significantly higher catalytic activity than Ru‐CTF‐900 for transfer hydrogenation of acetophenone.

## Graphical abstract

A simple yet novel strategy for the fabrication of sintering‐resistant mixed Ru single atoms and small nanoparticles (NPs) catalysts on N‐doped carbon with charge‐modulated CTF (covalent triazine framework) as the template. Through modification of the micro‐environment within the CTF architecture, the Ru precursor interacted on charged‐modulated CTF framework shows electrostatic repulsion and steric hindrance, protecting Ru atoms from severe sintering.

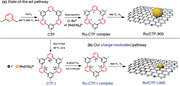

Metal‐organic frameworks (MOFs) and covalent organic frameworks (COFs) are important classes of crystalline porous organic architectures that have shown promising potential in different areas of application including gas sorption, energy storage, and conversion, catalysis, etc.^[^ ^1^, ^2^, ^3^ ^]^ Because of their high carbon contents and relative stability of the carbon framework, high‐temperature carbonization of MOFs or COFs has been identified as a facile synthetic strategy of obtaining high‐surface‐area porous carbon supports^[^ ^4^, ^5^, ^6^, ^7^ ^]^ or even various carbon‐supported metal nano‐catalysts, particularly from MOFs owing to their intrinsic metal‐organic hybrid nature.^[^ ^8^, ^9^, ^10^, ^11^ ^]^ Unfortunately, such a direct heat‐treatment process causes severe sintering of metal nanoparticles (NPs) at high temperatures due to high surface energy.^[^ ^12^ ^]^ The past few years have witnessed significant breakthroughs due to the rapid development of advanced synthetic strategies and characterization techniques, which ultimately led to the discovery of supported single‐atom catalysts (SACs).^[^ ^13^, ^14^, ^15^ ^]^ They usually display catalytic behavior that is remarkably different from those of large metal NPs while simultaneously maximizing metal efficiency, and they offer great potential for the realization of high catalytic activity and selectivity.^[^ ^16^, ^17^, ^18^ ^]^ However, the most advanced synthetic strategy of obtaining carbon‐supported metal SACs based on MOFs architecture, requires painstakingly grafting N‐rich organic linkers into the organic framework prior to pyrolysis, and subsequent etching process in an acidic medium post‐treatment step.^[^ ^19^, ^20^ ^]^

COFs is another class of crystalline porous organic architectures that have also been investigated as sacrificial templates for the fabrication of high‐surface‐area porous carbons due to their excellent porosity, great stability, and intrinsic rich heteroatoms.^[^ ^21^ ^]^ Covalent triazine frameworks (CTFs), a subclass of COFs, may have presented itself as highly viable candidates for this application, given its intrinsic high‐nitrogen content, and the ability to fabricate via facile high‐temperature ZnCl_2_‐mediated ionothermal processes.^[^ ^22^, ^23^, ^24^ ^]^ In fact, a series of CTF supported metal single atoms or clusters catalysts including Ru,^[^ ^25^, ^26^ ^]^ Pt,^[^ ^27^, ^28^ ^]^ Pd,^[^ ^29^ ^]^ have been reported for different catalytic applications, however, those studies all used the mixture of the metal precursor and CTF without further high‐temperature pyrolysis process to obtain stable N‐doped carbon‐supported SACs to the best of our knowledge. The major issue is that the direct thermal treatment of pristine CTFs architecture in presence of metal precursors usually results in severe aggregation of metal particles, resulting in unreasonable utilization of the nitrogen‐rich backbones.

In the assessment of these, we set to modify the micro‐environment within the CTF architecture with CH_3_I through charge modulation, in an attempt to develop N‐rich carbon‐supported SACs and nanoclusters that are based on modified CTF architecture (CTF‐I). Prior to that, the as‐synthesized CTF network was utilized as the support to disperse Ru particles via high‐temperature pyrolysis. It is important to state that the dispersed Ru suffered severe sintering of NPs during high‐temperature treatment at 900 °C, with a complete absence of isolated single Ru atoms. This indicates that the abundance of nitrogen heteroatom in CTF architecture does not necessarily translate to efficient immobilization of Ru at high pyrolysis temperature. The modified CTF architecture (CTF‐I), on the other hand, exhibited the interaction between CTF‐I and Ru precursor through robust electrovalent bonds and thus contributed toward a higher density of single Ru atoms, smaller Ru NPs, and ultimately higher catalytic activity toward catalytic transfer hydrogenation (CTH) of acetophenone. A large number of Ru SACs and ultra‐small Ru NPs were validated in Ru‐CTF‐I‐900 catalyst by atomic resolution high angle annular dark field transmission electron microscopy (TEM) and X‐ray absorption near‐edge spectroscopy. Powder X‐ray diffraction and TEM measurements revealed that the particle size of Ru in Ru‐CTF‐900 catalyst is significantly larger than that of Ru‐CTF‐I‐900. When compared with Ru‐CTF‐900, the N_2_ physisorption measurement of Ru‐CTF‐I‐900 shows a higher surface area and pore volume that provides more rest space for the Ru guests.

**Scheme** **1** summarizes the general synthetic strategy of Ru‐CTF‐900 catalyst, in which sacrificial CTF architecture was fabricated through classical polymerization of 2,6‐dicyanopyridine based on previously reported ZnCl_2_‐catalyzed ionothermal strategy at 400/600 °C.^[^ ^24^ ^]^ The detailed synthetic procedures are found in the Experimental Section. 2,6‐dicyanopyridine was chosen as the monomer because of the fact that trimerization condensation at temperatures higher than 400 °C affords in situ generations of the required rich N‐rich sites from the pyridine‐based backbones. Ru‐CTF‐900 catalyst was ultimately formed by the immersion of the as‐synthesized CTF architecture into a RuCl_3_ solution via traditional impregnation strategy,^[^ ^25^ ^]^ and subsequent pyrolysis of the impregnated network at 900 °C for 1 h in N_2_ atmosphere. **Figure** **1** shows the XRD pattern of the as‐obtained Ru‐CTF‐900 catalyst; as expected, six obvious diffraction peaks indexed to (100), (002), (101), (102), (110), and (103) of the hexagonal phase structure of Ru were observed in the pattern. The characteristic diffraction peaks are sharp suggesting the formation of large Ru NPs as also reflected in the TEM image (**Figure** **2**d and Figure S1, Supporting Information) and energy‐dispersive spectrum (EDS) mapping result (Figure S2, Supporting Information). Also, a new broad diffraction peak at ≈44° is visibly seen in the XRD pattern which may have resulted from the formation of another form of amorphous carbon during pyrolysis at high temperature. This observation is consistent with the XRD pattern of a pyrolyzed CTF architecture with no Ru loading shown in Figure S3a, Supporting Information. The XPS spectrum in **Figure** **3**c shows that the N1s profile can be deconvoluted into N_triazine_, N_cyano_, and N_graphitic_ at 398–399, 399–399.8, and 400–401 eV respectively, for the CTF architecture.^[^ ^30^ ^]^ The existence of N_triazine_ indicates the occurrence of cyclic trimerization of 2,6‐dicyanopyridine, as the retro‐trimerization of CTF led to the formation of uncondensed nitrile groups (N_cyano_).^[^ ^31^, ^32^ ^]^ The existence of N_graphitic_ is attributed to the presence of irreversible C—C coupling at high temperatures (600 °C). However, a significant decrease in nitrogen content (17.6% to 6.1%) was observed upon the pyrolysis of the Ru‐CTF complex at 900 °C to obtain Ru‐CTF‐900 catalysts as seen in Table S1, Supporting Information and Figure 3d. Meanwhile, the ratio of N_graphitic_ increases at the expense of N_cyano_ and N_triazine_, suggesting the formation of more graphitized fragments from cross‐linking/rearrangement reactions under thermal treatment at 900 °C.

**Scheme 1:**
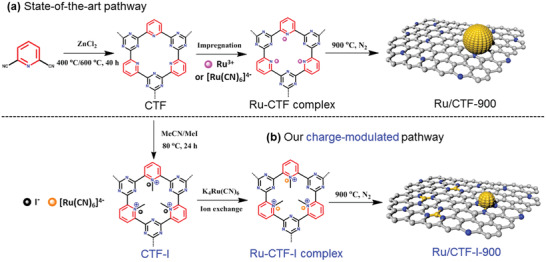
The schematic diagram for the synthesis of Ru‐CTF‐900 and Ru‐CTF‐I‐900 by conventional impregnation method and charge‐modulated strategy; for Ru/CTF‐900 and Ru‐CTF‐I‐900 samples, grey: C, blue: N, yellow: Ru.

**Figure 1:**
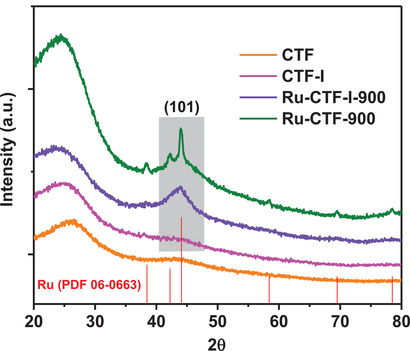
XRD patterns of CTF, CTF‐I, Ru‐CTF‐900, and Ru‐CTF‐I‐900 samples, respectively.

**Figure 2:**
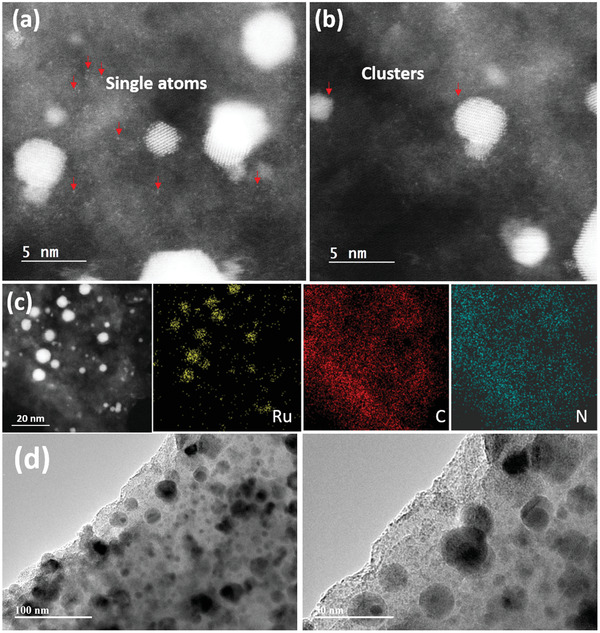
Atomic resolution HAADF images of a,b) Ru‐CTF‐I‐900; HAADF image and corresponding EDS mapping for Ru, C, and N of c) Ru‐CTF‐I‐900; d) TEM images of Ru‐CTF‐900.

**Figure 3:**
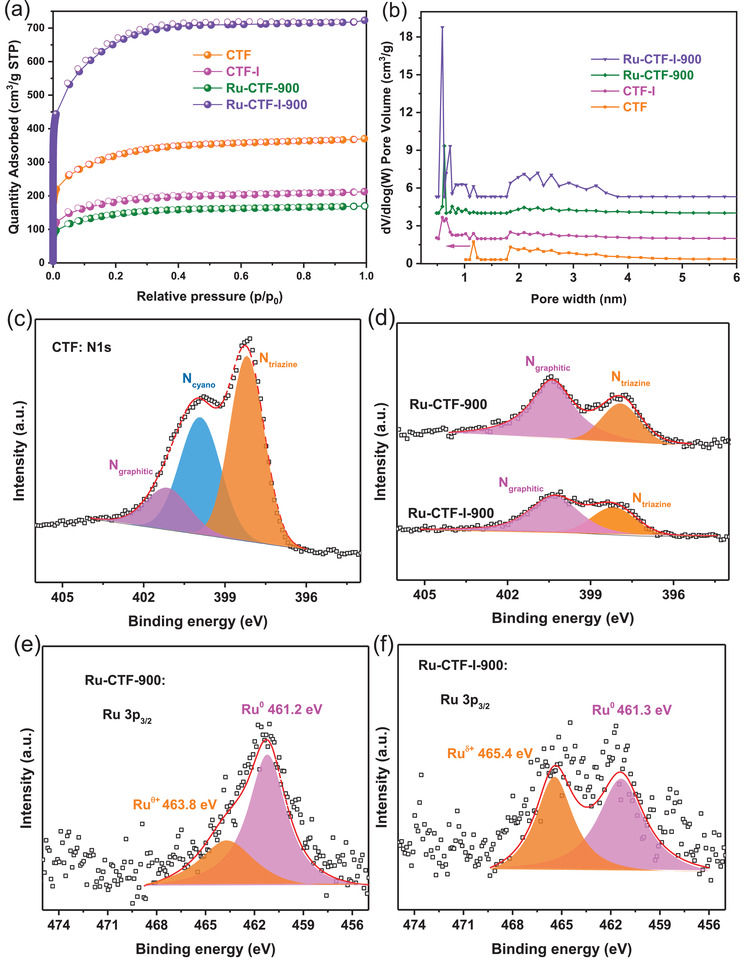
a) N_2_ adsorption‐desorption isotherms and the b) corresponding pore size distribution of CTF, CTF‐I, Ru‐CTF‐900, and Ru‐CTF‐I‐900; c) N1s XPS spectra for CTF; d) Ru‐CTF‐900 and Ru‐CTF‐I‐900; Ru3p_3/2_ XPS spectra for e) Ru‐CTF‐900 and f) Ru‐CTF‐I‐900.

Unfortunately, the N_2_ adsorption‐desorption isotherm of Ru‐CTF‐900 (Figure 3a) indicates a rearrangement of Ru‐CTF complex and consequent collapse of porous structures upon pyrolysis. This is reflected by the profound difference in surface area between Ru‐CTF‐900 (507 m^2^ g^−1^) and CTF (1058 m^2^ g^−1^, Table S2, Supporting Information). For the XPS measurement, due to the fact that Ru3d_3/2_ and partial Ru3d_5/2_ peaks overlap with that of C1s, the Ru 3p_3/2_ region was used to investigate the surface electronic structures of Ru.^[^ ^33^ ^]^ The XPS profile of Ru‐CTF‐900 shows a mixture of components of metallic Ru^0^ and oxidized Ru*^*θ*^* ^+^ species but predominantly metallic Ru^0^ (Figure 2e). The XANES spectrum of Ru‐CTF‐900 in **Figure** **4**a exhibits a very slightly higher energy absorption edge than the Ru foil. Also, the X‐ray absorption fine structure (EXAFS) profile of Ru‐CTF‐900 matches perfectly with the Ru foil, indicating that Ru^0^ is the dominant component of Ru‐CTF‐900. These observations are both consistent with the XPS results. A high Ru—Ru coordination number of 7.2 was determined for the Ru‐CTF‐900 catalyst. It, therefore, follows that immobilization of Ru NPs on pristine CTF is quite difficult to maintain under high‐temperature pyrolysis. Also, this constitutes the major obstacle limiting the synthesis of single metal atoms or even small metal clusters on CTFs‐derived carriers.

**Figure 4:**
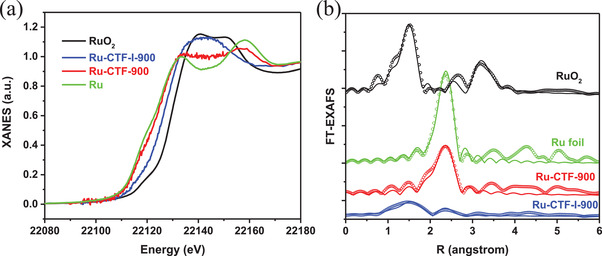
a) Ru K‐edge XANES and b) the corresponding EXAFS spectra for Ru foil, Ru‐CTF‐900, and Ru‐CTF‐I‐900 samples.

To overcome this barrier, we modified the micro‐environment within the CTF architecture to form a CTF‐I network. This charge‐modulated CTF‐I template was prepared via a facile nucleophilic reaction between iodomethane and the pyridine nitrogen of CTF in acetonitrile medium (Scheme 1). The I^−^ ion of the modified CTF‐I carrier was replaced by the [Ru(CN)_6_]^4−^ precursor by an ion‐exchange reaction to form the robust Ru‐carrier electrovalent bonds.^[^ ^34^ ^]^ Following thermal treatment at 900 °C in the N_2_ atmosphere, ultra‐small Ru particles and abundant single atoms were found on the resultant Ru‐CTF‐I‐900 catalyst (Scheme 1). The decrease in N_2_ adsorption amount (Figure 3a), pore width (Figure 3b), surface area, and pore volume (Table S2, Supporting Information) in CTF‐I carrier relative to CFT architecture, provides direct evidence of the occurrence of nucleophilic reaction between iodomethane and the pyridine nitrogen of CTF. Analyses of the XPS profiles of CTF, CTF‐I, and I3d spectrum of CTF‐I provide additional evidence of a successful modification of the CTF network (Figures S4 and S5, Supporting Information). The as‐synthesized CTF‐I has iodine content of 1.9 wt% and similar surface C/N concentration with CTF (Table S2, Supporting Information), indicating that the presence of iodide ions in the CTF network merely reduces the pore size of the CTF channel but does not change the framework.^[^ ^30^ ^]^ Additionally, loss of N atoms is also observed during the pyrolysis of Ru‐CTF‐I complex with a significant increase in the volume of the mesoporous and microporous channels, Figure 3b, Table S2, Supporting Information), underlying the importance of charge modulation in pore reconstruction. Three kinds of N centers between CTF and CTF‐I also retain similar percentages (Figure 3c and Figures S6 and S7, Supporting Information).

XRD pattern of the as‐synthesized Ru‐CTF‐I‐900 catalyst was acquired to gain some crystallographic information on the structure. Unlike Ru‐CTF‐900, the diffraction pattern of Ru‐CTF‐I‐900 (Figure 1) surprisingly showed no characteristic peak belonging to the hexagonal phase structure of Ru; this suggests that the modified CTF‐I template sufficiently provided the required resistance to sintering of Ru particles during high‐temperature pyrolysis, which is essential for the production of ultra‐small Ru particles or single atoms. Atomic resolution high angle annular dark‐field (HAADF) TEM images of Ru‐CTF‐I‐900 in (Figure 2a,b, Figure S8, Supporting Information) show clear bright spots as uniform Ru single atoms and small NPs (<5 nm). The EDS elemental maps of a different area of the sample (Figure 2c) further confirms the uniform distribution of Ru single atoms and small NPs on the N‐rich carbon carrier. To further underscore the unique ability of our novel CTF‐I template, activated carbon (AC) as a classical carbon material was used to disperse Ru using the same method. The XRD pattern of the as‐synthesized Ru‐AC‐900 exhibits obvious sharp diffraction peaks ascribed to Ru NPs (Figure S9, Supporting Information), highlighting the intrinsic inability of “naked” carriers such as CTF or AC in the stabilization of single atoms during via facile pyrolysis. The Ru loading of Ru‐CTF‐900 and Ru‐CTF‐I‐900 was determined by inductively coupled plasma optical emission spectroscopy; the results are presented in Table S2, Supporting Information. Considering the fact that Ru‐CTF‐900 (1.2%) and Ru‐CTF‐I‐900 (0.8%) contain similar Ru loading, we believe that our charge modulation strategy has a profound influence on the controllable growth of Ru under high temperature.

To investigate the origin of the sintering‐resistant ability of the CTF‐I template, XPS, and XANES profiles of the sample were acquired. The XPS profile of Ru‐CTF‐I‐900 (Figure 3f) shows a higher percentage of oxidized Ru*^*δ*^* ^+^ than Ru^0^ and at a higher binding energy region as compared with Ru*^*θ*^* ^+^ in the Ru‐CTF‐900 profile. This more electron‐loss phenomenon is possibly due to the stronger coupling interactions that existed between Ru and the nitrogen heteroatoms of the CTF carrier.^[^ ^35^ ^]^ The XANES profiles in Figure 4a shows an obvious higher energy absorption edge of Ru‐CTF‐I‐900 as compared to Ru‐CTF‐900 and Ru foil, additionally collaborating the observed higher electron‐deficiency of Ru*^*δ*^* ^+^ seen in the XPS profiles. The EXAFS spectra of Ru‐CTF‐I‐900 show a weak Ru—Ru peak as compared to Ru‐CTF‐900 (Figure 4b), and a dominant peak at ≈1.5 Å (with 1.93 Å bond lengths in Table S3, Supporting Information) attributed to strong Ru—N interactions. The coordination number of the Ru—N interaction in our novel Ru‐CTF‐I‐900 catalyst is ≈3 based on the R space fitting results (Table S3, Supporting Information), which is consistent with the fact the energy absorption edge of Ru in Ru‐CTF‐I‐900 is lower than RuO_2_. This perhaps is the origin of the stabilization of Ru single atoms and clusters on the carrier template (Table S3, Supporting Information). Although ≈5 nm particle size Ru are present in Ru‐CTF‐I‐900 sample (based on TEM image in Figure 2a), the Ru—Ru coordination number is only 1.3, which indicates that a vast majority of Ru in our Ru‐CTF‐I‐900 catalyst exist as single atoms in combination with a very small percentage of ultra‐fine Ru NPs. Notably, substituting RuCl_3_ with K_4_[Ru(CN)_6_] as the metal precursor to obtain Ru‐CTF‐900′ following pyrolysis at 900 °C also produced a diffraction pattern that shows the characteristic sharp Ru peaks (Figure S10, Supporting Information), further collaborating the uniqueness of our charge modulation‐mediated strategy.

To explain the underlying cause that Ru‐CTF‐I‐900 shows a much smaller Ru size than that of Ru‐CTF‐900, density functional theory calculation of their CTF precursor is performed by using Gaussian 09 software. Cluster models were built, as shown in **Figure** **5**. In the model, the RuCl_3_ unit is captured by the CTF motif, where Ru coordinates two neutral nitrogen atoms with a binding energy of −81.5 kcal mol^−1^ and bond lengths of 2.178 and 2.207 Å, respectively. These nitrogen atoms can further coordinate with the RuCl_3_ unit on the other side of the motif, with a binding energy of −30.2 kcal mol^−1^. Besides, the RuCl_3_ unit coordinated with the motif is capable to form dimer by connecting one more RuCl_3_ via chloride bridges, with a binding energy of −53.7 kcal mol^−1^. Herein, these Ru atoms tend to aggregate into clusters during high‐temperature pyrolysis. On the other hand, the positively charged CTF‐I framework attracts the large Ru(CN)_6_^4−^ anion by electrostatic interaction. Ru center is apart from nitrogen atoms in the motif. The binding energy between the motif and K_4_[Ru(CN)_6_] is −22.0 kcal mol^−1^. The electrostatic repulsion and steric hindrance inhibit further binding with more Ru‐containing anions, which may be responsible for the formation of the evenly dispersed Ru atoms and clusters after high‐temperature pyrolysis.

**Figure 5:**
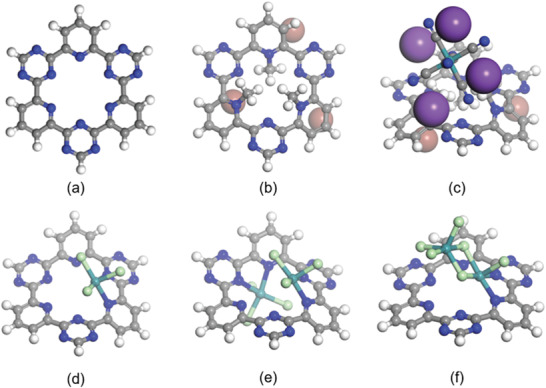
Optimized structures of a) CTF motif; b) CTF‐I motif; c) CTF‐I‐K_4_[Ru(CN)_6_]; d) CTF‐RuCl_3_; e) CTF‐2(RuCl_3_); f) CTF‐(RuCl_3_)_2_.

Ru SACs and small NPs have been synthesized and evaluated for both thermocatalysis and electrocatalysis applications.^[^ ^36^, ^37^ ^]^ CTH of acetophenone is considered an important probe reaction in both industrial and academic settings.^[^ ^38^, ^39^, ^40^ ^]^ Although homogeneous Ru‐based complexes have shown promising potential for this application,^[^ ^41^ ^]^ the catalytic activity of heterogeneous supported Ru catalyst, particularly in a base‐free medium still requires improvement. The catalytic performance of the synthesized Ru‐CTF‐900 and Ru‐CTF‐I‐900 catalysts was compared for base‐free CTH of acetophenone at different reaction conditions. The obtained conversion and yields are summarized in **Table** **1**. 1‐phenylethanol is the main product of this conversion process with <1% styrene observed as the over‐hydrogenated byproduct between 120–140 °C of reaction temperature, over Ru‐CTF‐I‐900 catalyst. Furthermore, the Ru‐CTF‐I‐900 catalyst gave a higher conversion of acetophenone and yield of 1‐phenylethanol than the Ru‐CTF‐900 catalyst for all reaction temperatures and times under review (Table 1). For example, Ru‐CTF‐I‐900 catalyst afforded nearly a sevenfold yield of 1‐phenylethanol than Ru‐CTF‐900 catalyst at 100 °C for 24 h (entry 3,4). Under optimized reaction conditions of temperature and time (140 °C, 24 h), a 100% conversion of acetophenone and 99% yield of 1‐phenylethanol was observed over the Ru‐CTF‐I‐900 (entry 7) catalyst as compared to 90% yield of 1‐phenylethanol realized over the Ru‐CTF‐900 catalyst (entry 8). Additionally, Ru‐CTF‐I‐900 catalyst exhibits good catalytic stability for three repeated use (Table S4, Supporting Information), and no diffraction peaks ascribed to larger Ru clusters can be found on used Ru‐CTF‐I‐900 catalyst (Figure S11, Supporting Information). The superior performance of the Ru‐CTF‐I‐900 catalyst is consistent with its unique features. Therefore, our charge‐modulated synthetic strategy could potentially pave the way to many possibilities of fabricating sintering‐resistant ultra‐small metal NPs or single atoms on crystalline porous organic architectures, which might contribute to higher catalytic performance for most catalytic systems.

**Table 1:** Ruthenium‐catalyzed transfer hydrogenation of acetophenone

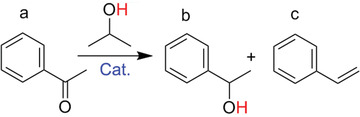						
Entry	Catalysts	T [°C]	Time [h]	Conversion [%]	Yield (b) [%]	Yield (c) [%]
1	Ru‐CTF‐I‐900	100	12	41.3	37.8	–
2	Ru‐CTF‐900	100	12	8.6	4.3	–
3	Ru‐CTF‐I‐900	100	24	57.0	55.1	–
4	Ru‐CTF‐900	100	24	11.2	7.1	–
5	Ru‐CTF‐I‐900	120	24	76.0	74.8	0.6
6	Ru‐CTF‐900	120	24	32.2	28.1	–
7	Ru‐CTF‐I‐900	140	24	100	99	0.8
8	Ru‐CTF‐900	140	24	92	90	–

In summary, traditional CTF and charge‐modulated CTF‐I architectures were synthesized and subsequently utilized as N‐rich sacrificial templates for the preparation of Ru‐CTF‐900 and Ru‐CTF‐I‐900 catalysts respectively, via facile pyrolysis at 900 °C in N_2_. The dispersed Ru on unmodified CTF architecture suffers severe sintering during high‐temperature pyrolysis at 900 °C, indicating that high nitrogen contents of the CTF architecture do not necessarily translate to efficient immobilization of Ru at high temperature. Through modification of the micro‐environment within the CTF architecture, the CTF‐I and Ru precursor are interacted by robust electrovalent bonds and then induces the electrostatic repulsion and steric hindrance, thus contributing toward the high density of single Ru atoms and even smaller Ru NPs after pyrolysis. A Ru—Ru coordination number of only 1.3 was observed in our novel Ru‐CTF‐I‐900 catalyst which exhibited significantly higher catalytic activity than the unmodified Ru‐CTF‐900 counterpart toward transfer hydrogenation of acetophenone in the absence of a base. Therefore, our charge‐modulated synthetic strategy could potentially pave the way to many possibilities of fabricating sintering‐resistant ultra‐small metal NPs or single atoms on porous organic architectures, which might contribute to higher catalytic performance for most catalytic applications.

## Experimental Section

##### Synthesis of CTF and Ru‐CTF‐900

CTF was fabricated in sealed quartz tubes through ionothermal synthesis using 2,6‐dicyanopyridine (258 mg) as the monomer and anhydrous ZnCl_2_ (2.72 g) as both solvent and catalyst based on the previous study.^[^ ^42^ ^]^ After thermal treatment at 400 °C for 20 h and 600 °C for another 20 h, the resultant black powder was ground and then washed three times each with H_2_O, HCl, and acetone to remove ZnCl_2_. The final product was obtained by a simple drying process. The residual concentration of Zn as determined by ICP‐OES analysis in the CTF was only 1.6% (Table S2, Supporting Information). Ru/CTF‐900 was synthesized by incipient wetness impregnation with an aqueous solution of RuCl_3_. Briefly, an aqueous solution of Ru^3+^ was uniformly dropped into the CTF carrier and then sonicated for 30 min. The resultant mixture was dried overnight under reduced pressure at 50 °C and calcined at 900 °C in N_2_ atmosphere for 1 h to obtain Ru‐CTF‐900. The Ru weight loading of Ru‐CTF‐900 as determined by ICP‐OES analysis was ≈1.2 wt% (Table S1, Supporting Information). Ru‐CTF‐900′ was synthesized in a similar fashion using K_4_[Ru(CN)_6_] as the Ru source. To eliminate the influence of Ru loading, the XRD pattern of Ru‐CTF‐900 with lower Ru loading of ≈0.6 wt% was collected in Figure S3b, Supporting Information, and the obvious diffraction peaks ascribed to metallic Ru can also be seen.

##### Synthesis of CTF‐I and Ru‐CTF‐I‐900

In a typical experiment, CTF (0.2 g) and acetonitrile (CH_3_CN: 100 mL) were mixed in a three‐necked Schlenk flask. Following nitrogen purge for ≈30 min, CH_3_I (0.5 mL) was injected into the flask and then refluxed at 80 °C for 24 h. The solid was recovered by filtration and then washed three times each with H_2_O, CH_3_CN, ethanol, and acetone in that order. CTF‐I was obtained after overnight drying of the sample under reduced pressure at 50 °C. Ru/CTF‐I‐900 was synthesized by ion‐exchange reaction using an aqueous solution of K_4_[Ru(CN)_6_] as the Ru source. Briefly, an aqueous solution of [Ru(CN)_6_]^4−^ was uniformly mixed with CTF‐I carrier and then sonicated for 30 min. The resultant mixture was stirred for ≈12 h to ensure complete ion exchange. The resultant mixture was dried overnight under reduced pressure at 50 °C and calcined at 900 °C in an N_2_ atmosphere for 1 h to obtain Ru‐CTF‐I‐900. The Ru weight loading as determined by ICP‐OES analysis was ≈0.8 wt% (Table S1, Supporting Information).

## Conflict of Interest

The authors declare no conflict of interest.

1

Y.
Peng
, 
M.
Zhao
, 
B.
Chen
, 
Z.
Zhang
, 
Y.
Huang
, 
F.
Dai
, 
Z.
Lai
, 
X.
Cui
, 
C.
Tan
, 
H.
Zhang
, Adv. Mater.
2018, 30, 1705454.10.1002/adma.201705454291346772

G.
Maurin
, 
C.
Serre
, 
A.
Cooper
, 
G. J. C. S. R.
Férey
, Chem. Soc. Rev.
2017, 46, 3104.2856109010.1039/c7cs90049j3

K.
Chen
, 
Z.
Sun
, 
R.
Fang
, 
Y.
Shi
, 
H. M.
Cheng
, 
F.
Li
, Adv. Funct. Mater.
2018, 28, 1707592.4

B.
Liu
, 
H.
Shioyama
, 
T.
Akita
, 
Q.
Xu
, J. Am. Chem. Soc.
2008, 130, 5390.1837683310.1021/ja71061465

H.‐L.
Jiang
, 
B.
Liu
, 
Y.‐Q.
Lan
, 
K.
Kuratani
, 
T.
Akita
, 
H.
Shioyama
, 
F.
Zong
, 
Q.
Xu
, J. Am. Chem. Soc.
2011, 133, 11854.2175178810.1021/ja203184k6

X.
Lan
, 
Y.
Li
, 
C.
Du
, 
T.
She
, 
Q.
Li
, 
G.
Bai
, Chem. ‐ Eur. J.
2019, 25, 8560.3095010010.1002/chem.2019005637

X.
Zhang
, 
G.
Zhu
, 
M.
Wang
, 
J.
Li
, 
T.
Lu
, 
L.
Pan
, Carbon
2017, 116, 686.8

M.
Zhang
, 
Q.
Dai
, 
H.
Zheng
, 
M.
Chen
, 
L.
Dai
, Adv. Mater.
2018, 30, 1705431.10.1002/adma.201705431293498419

Y.
Yusran
, 
D.
Xu
, 
Q.
Fang
, 
D.
Zhang
, 
S.
Qiu
, Microporous Mesoporous Mater.
2017, 241, 346.10

A.
Li
, 
K.
Shen
, 
J.
Chen
, 
Z.
Li
, 
Y.
Li
, Chem. Eng. Sci.
2017, 166, 66.11

N. L.
Torad
, 
M.
Hu
, 
S.
Ishihara
, 
H.
Sukegawa
, 
A. A.
Belik
, 
M.
Imura
, 
K.
Ariga
, 
Y.
Sakka
, 
Y.
Yamauchi
, Small
2014, 10, 2096.2461068410.1002/smll.20130291012

B.
Tang
, 
W.
Song
, 
E.
Yang
, 
X.
Zhao
, RSC Adv.
2017, 7, 1531.13

B.
Qiao
, 
A.
Wang
, 
X.
Yang
, 
L. F.
Allard
, 
Z.
Jiang
, 
Y.
Cui
, 
J.
Liu
, 
J.
Li
, 
T.
Zhang
, Nat. Chem.
2011, 3, 634.2177898410.1038/nchem.109514

J.
Liu
, ACS Catal.
2016, 7, 34.15

A.
Wang
, 
J.
Li
, 
T.
Zhang
, Nat. Rev. Chem.
2018, 2, 65.16

L.
Wang
, 
H.
Li
, 
W.
Zhang
, 
X.
Zhao
, 
J.
Qiu
, 
A.
Li
, 
X.
Zheng
, 
Z.
Hu
, 
R.
Si
, 
J.
Zeng
, Angew. Chem., Int. Ed.
2017, 56, 4712.10.1002/anie.2017010892837095517

H.
Zhang
, 
G.
Liu
, 
L.
Shi
, 
J.
Ye
, Adv. Energy Mater.
2018, 8, 1701343.18

X.
Fang
, 
Q.
Shang
, 
Y.
Wang
, 
L.
Jiao
, 
T.
Yao
, 
Y.
Li
, 
Q.
Zhang
, 
Y.
Luo
, 
H. L.
Jiang
, Adv. Mater.
2018, 30, 1705112.10.1002/adma.2017051122931587119

X.
Wang
, 
W.
Chen
, 
L.
Zhang
, 
T.
Yao
, 
W.
Liu
, 
Y.
Lin
, 
H.
Ju
, 
J.
Dong
, 
L.
Zheng
, 
W.
Yan
, 
X.
Zheng
, 
Z.
Li
, 
X.
Wang
, 
J.
Yang
, 
D.
He
, 
Y.
Wang
, 
Z.
Deng
, 
Y.
Wu
, 
Y.
Li
, J. Am. Chem. Soc.
2017, 139, 9419.2866113010.1021/jacs.7b0168620

Q.‐L.
Zhu
, 
W.
Xia
, 
L.‐R.
Zheng
, 
R.
Zou
, 
Z.
Liu
, 
Q.
Xu
, ACS Energy Lett.
2017, 2, 504.21

Y.‐B.
Huang
, 
P.
Pachfule
, 
J.‐K.
Sun
, 
Q.
Xu
, J. Mater. Chem. A
2016, 4, 4273.22

K.
Wang
, 
L. M.
Yang
, 
X.
Wang
, 
L.
Guo
, 
G.
Cheng
, 
C.
Zhang
, 
S.
Jin
, 
B.
Tan
, 
A.
Cooper
, Angew. Chem., Int. Ed.
2017, 56, 14149.10.1002/anie.201708548PMC56986982892668823

M.
Liu
, 
K.
Jiang
, 
X.
Ding
, 
S.
Wang
, 
C.
Zhang
, 
J.
Liu
, 
Z.
Zhan
, 
G.
Cheng
, 
B.
Li
, 
H.
Chen
, 
S.
Jin
, 
B.
Tan
, Adv. Mater.
2019, 31, 1807865.10.1002/adma.2018078653092070924

P.
Kuhn
, 
M.
Antonietti
, 
A.
Thomas
, Angew. Chem., Int. Ed.
2008, 47, 3450.10.1002/anie.2007057101833087825

J.
Artz
, 
S.
Mallmann
, 
R.
Palkovits
, ChemSusChem
2015, 8, 672.2558631210.1002/cssc.20140307826

S.
Yamaguchi
, 
K.
Kamiya
, 
K.
Hashimoto
, 
S.
Nakanishi
, Chem. Commun.
2017, 53, 10437.10.1039/c7cc05841a2888477727

K.
Kamiya
, 
R.
Kamai
, 
K.
Hashimoto
, 
S.
Nakanishi
, Nat. Commun.
2014, 5, 5040.2524221410.1038/ncomms6040PMC419911228

J.
Kim
, 
H. E.
Kim
, 
H.
Lee
, ChemSusChem
2018, 11, 104.2889531510.1002/cssc.20170130629

T.
He
, 
L.
Liu
, 
G.
Wu
, 
P.
Chen
, J. Mater. Chem. A
2015, 3, 16235.30

Y.
Li
, 
S.
Zheng
, 
X.
Liu
, 
P.
Li
, 
L.
Sun
, 
R.
Yang
, 
S.
Wang
, 
Z. S.
Wu
, 
X.
Bao
, 
W. Q.
Deng
, Angew. Chem., Int. Ed.
2018, 57, 7992.10.1002/anie.2017111692913506331

S.
Kundu
, 
W.
Xia
, 
W.
Busser
, 
M.
Becker
, 
D. A.
Schmidt
, 
M.
Havenith
, 
M.
Muhler
, Phys. Chem. Chem. Phys.
2010, 12, 4351.2040770610.1039/b923651a32

Y.
Zhao
, 
K.
Yao
, 
B.
Teng
, 
T.
Zhang
, 
Y.
Han
, Energy Environ. Sci.
2013, 6, 3684.33

K.
Yang
, 
P.
Xu
, 
Z.
Lin
, 
Y.
Yang
, 
P.
Jiang
, 
C.
Wang
, 
S.
Liu
, 
S.
Gong
, 
L.
Hu
, 
Q.
Chen
, Small
2018, 14, e1803009.3035055310.1002/smll.20180300934

S.
Ding
, 
C.
Tian
, 
X.
Zhu
, 
H.
Wang
, 
H.
Wang
, 
C. W.
Abney
, 
N.
Zhang
, 
S.
Dai
, Chem. Commun.
2018, 54, 5058.10.1039/c8cc02966k2972687135

C.
Lei
, 
Y.
Wang
, 
Y.
Hou
, 
P.
Liu
, 
J.
Yang
, 
T.
Zhang
, 
X.
Zhuang
, 
M.
Chen
, 
B.
Yang
, 
L.
Lei
, Energy Environ. Sci.
2019, 12, 149.36

Z.
Geng
, 
Y.
Liu
, 
X.
Kong
, 
P.
Li
, 
K.
Li
, 
Z.
Liu
, 
J.
Du
, 
M.
Shu
, 
R.
Si
, 
J.
Zeng
, Adv. Mater.
2018, 30, 1803498.10.1002/adma.2018034983009585537

C.
Xiao
, 
Z.
Cai
, 
T.
Wang
, 
Y.
Kou
, 
N.
Yan
, Angew. Chem., Int. Ed.
2008, 47, 746.10.1002/anie.2007034811806711138

P. O.
Lagaditis
, 
A. J.
Lough
, 
R. H.
Morris
, J. Am. Chem. Soc.
2011, 133, 9662.2162715210.1021/ja202375y39

R. B.
Nasir Baig
, 
R. S.
Varma
, ACS Sustainable Chem. Eng.
2013, 1, 805.10.1021/acssuschemeng.7b00772PMC61454833024594140

D. J.
Braden
, 
R.
Cariou
, 
J. W.
Shabaker
, 
R. A.
Taylor
, Appl. Catal., A
2019, 570, 367.41

S.
Dayan
, 
N.
Ozpozan Kalaycioglu
, 
J. C.
Daran
, 
A.
Labande
, 
R.
Poli
, Eur. J. Inorg. Chem.
2013, 2013, 3224.42

J.
Roeser
, 
K.
Kailasam
, 
A.
Thomas
, ChemSusChem
2012, 5, 1793.2289934310.1002/cssc.201200091

## Supporting information

Supporting Information
