# Supplementary material for: Sacrificial Synthesis of Supported Ru Single Atoms and Clusters on N‐doped Carbon Derived from Covalent Triazine Frameworks: A Charge Modulation Approach
Source: Adv Sci (Weinh). 2020 Dec 20;8(3):2001493. doi: 10.1002/advs.202001493 (PMC7856886; doi:10.1002/advs.202001493)
Supplement: Supplementary file 1 — Supporting Information [file ADVS-8-2001493-s001.pdf]

WILEY-VCH

© 2020 Wiley-VCH GmbH

ADVANCED SCIENCE
Open Access

Supporting Information

for *Adv. Sci.*, DOI: 10.1002/advs.advs202001493

Sacrificial Synthesis of Supported Ru Single Atoms and Clusters on N-doped Carbon Derived from Covalent Triazine Frameworks: A Charge Modulation Approach

Zihao Zhang, Siyu Yao, Xiaobing Hu, Francis Okejiri, Kun He, Pingying Liu, Ziqi Tian, Vinayak P. Dravid, Jie Fu, Xiang Zhu* and Sheng Dai**

Supporting Information

Sacrificial Synthesis of Supported Ru Single Atoms and Clusters on N-doped Carbon Derived from Covalent Triazine Frameworks: A Charge Modulation Approach

Zihao Zhang, Siyu Yao, Xiaobing Hu, Francis Okejiri, Kun He, Pingying Liu, Ziqi Tian, Vinayak P. Dravid, Jie Fu, Xiang Zhu* and Sheng Dai**

Dr. Z. Zhang, Dr. S. Yao, Dr. J. Fu
Key Laboratory of Biomass Chemical Engineering of Ministry of Education, College of Chemical and Biological Engineering, Zhejiang University, Hangzhou 310027, China
E-mail: jiefu@zju.edu.cn

Dr. X. Zhu
State Key Laboratory for Oxo Synthesis and Selective Oxidation, Suzhou Research Institute of Lanzhou Institute of Chemical Physics, Chinese Academy of Sciences, Lanzhou 730000, China
E-mail: xiang@licp.cas.cn

Dr. Z. Zhang, F. Okejiri, Prof. S. Dai
Department of Chemistry, The University of Tennessee, Knoxville, TN 37996, USA; Chemical Sciences Division, Oak Ridge National Laboratory, Oak Ridge, TN 37831, USA
E-mail: dais@ornl.gov

Dr. X. Hu, Dr. K. He, Prof. V. P. Dravid
Department of Materials Science and Engineering, Northwestern University, Evanston, IL 60208, USA; The NU*ANCE* Center, Northwestern University, Evanston, IL 60208, USA

P. Liu, Dr. Z. Tian
Ningbo Institute of Materials Technology and Engineering, Chinese Academy of Sciences, Ningbo 315201, Zhejiang, China

Dr. J. Fu
Institute of Zhejiang University – Quzhou, 78 Jiuhua Boulevard North, Quzhou 324000, Zhejiang, China

Experimental Section

Chemicals: All chemicals and solvents are commercially available from local suppliers, and used without further purification. Deionized water was obtained in our laboratory.

Characterizations: powder X-rays diffraction (PXRD) was carried out on a PANalytical Empyrean diffractometer operated at 45 kV and 40 mA with the canning rate of 0.02° per step. N_2 adsorption-desorption isotherms were analyzed on a 3Flex Micromeritics adsorption instrument. Prior to analysis, all samples were activated at 160 °C for 8 h. High angle annular dark-field (HAADF) imaging and associated energy dispersive spectrum (EDS) analyses were performed on ARM 200CF equipped with a probe corrector and dual solid silicon drift (SSD) detectors. The TEM is operated at 200 kV. Elemental analysis of all samples was performed by inductively coupled plasma atomic emission spectroscopy (ICP-AES) by the Optima 2100 DV spectrometer (PerkinElmer Corporation). X-ray photoelectron spectroscopy (XPS) was conducted in ESCALab 250Xi (Thermo Scientific, USA), and the obtained data were analyzed by Casa XPS software. The Ru K edge X-ray absorption spectra were collected at the 8-ID (ISS) beamline of the National Synchrotron Light Source II (NSLS-II) of the Brookhaven National Laboratory (BNL). The samples were smeared and sealed in the Kapton^{TM} tape and collected using the fluorescence mode using a passivated implanted planar silicon (PIPS) detector. The collected spectra were treated and fitted using the Ifeffit software package.

Catalytic transfer hydrogenation (CTH) of acetophenone: A certain amount of acetophenone (100 mg) and catalysts (10 mg) together with 3.5 mL isopropanol were added simultaneously into 8 mL stainless steel autoclave. The sealed autoclave was purged three times with N_2 to remove air. After reaction at required reaction temperature and time, the reactor was cooled down by cold water to room temperature. Thereafter, the liquid products were collected and analyzed by gas chromatography.

Calculation details

All DFT calculations were performed using the Gaussian 09 program package.[1] The geometry optimizations were carried out by using B3LYP hybrid functional with LANL2dz pseudopotentials for Ru and I, and 6-31G(d) basis sets for other elements. The binding strengths were evaluated based on binding energies by using M06-2X hybrid functional with SDD basis sets for Ru and I, and 6-311++G(d, p) for other elements, respectively. The basis set superposition error (BSSE) was computed by the counterpoise method.[2,3]

Table S1. XPS elemental analyses results

Samples	C	N	I	Ru	Cl
CTF	80.8	17.6	~	~	1.6
CTF-I	78.7	19.3	1.9	~	~
Ru-CTF-900	93.1	6.1	~	0.8	~
Ru-CTF-I-900	92.7	6.6	~	0.6	~

The composition ratio (atomic %) for CTF, CTF-I, Ru-CTF-900 and Ru-CTF-I-900 samples.

Table S2. Summary of pore parameter for CTF, CTF-I, Ru-CTF-900 and Ru-CTF-I-900 samples using N_2 adsorption-desorption isotherms at 77 K.

Samples	S_{BET} ($m^2\ g^{-1}$)[a]	Pore volume ($cm^3\ g^{-1}$)[b]	Average pore size (nm)[c]	Ru loading (wt %)[d]
CTF	1058	0.57	2.1	~
CTF-I	608	0.33	2.1	~
Ru-CTF-900	507	0.26	2.1	1.2
Ru-CTF-I-900	2212	1.12	2.0	0.8

[a]Specific surface area calculated using the BET equation.

[b]Single point adsorption total pore volume of pores.

[c]Adsorption average pore diameter (4V/A by BET).

[d]Determined by ICP-OES result.

Table S3. R space fitting results from Ru K-edge for Ru-CTF-I-900, Ru-CTF-900 and Ru foil samples

Samples	Path	Coordination number	Bond length (Å)	$\sigma^2/10^{-3}$
Ru-CTF-I-900	Ru-N	2.9+/-0.9	1.93+/-	4.0
	Ru-O	1.0+/-0.5	2.08+/-	4.0
	Ru-Ru	1.3+/-0.2	2.67+/-	6.0
Ru-CTF-900	Ru-Ru	7.2+/-2.6	2.67+/-	4.0
Ru foil	Ru-Ru	S02=0.86	2.677	

Table S4. The reuse performance of Ru-CTF-I-900 and Ru-CTF-900 on transfer hydrogenation of acetophenone

Usage time	Catalysts	T/°C	Time/h	Conversion/%	Yield of 1-phenylethanol/%
1	Ru-CTF-I-900	120	24	76.0	74.8
2	Ru-CTF-I-900	120	24	76.7	75.3
3	Ru-CTF-I-900	120	24	74.9	73.4
1	Ru-CTF-900	120	24	32.2	28.1
2	Ru-CTF-900	120	24	31.3	28.0
3	Ru-CTF-900	120	24	30.9	27.4

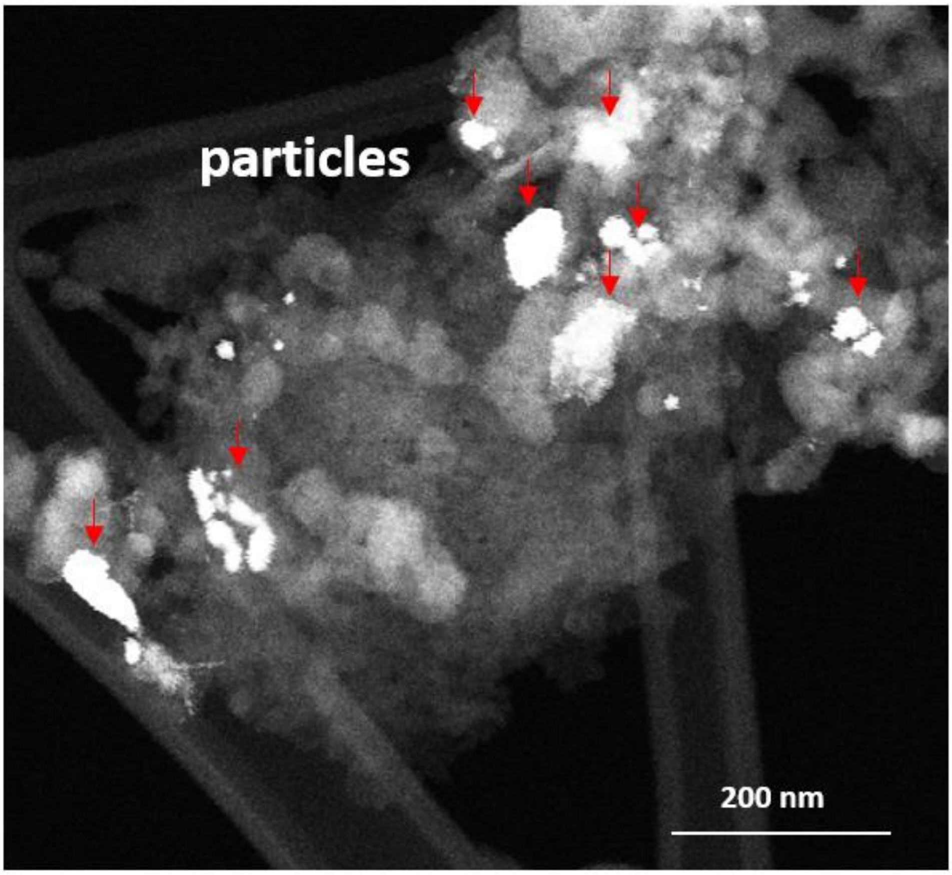

Figure S1. HAADF TEM image of Ru-CTF-900 sample.

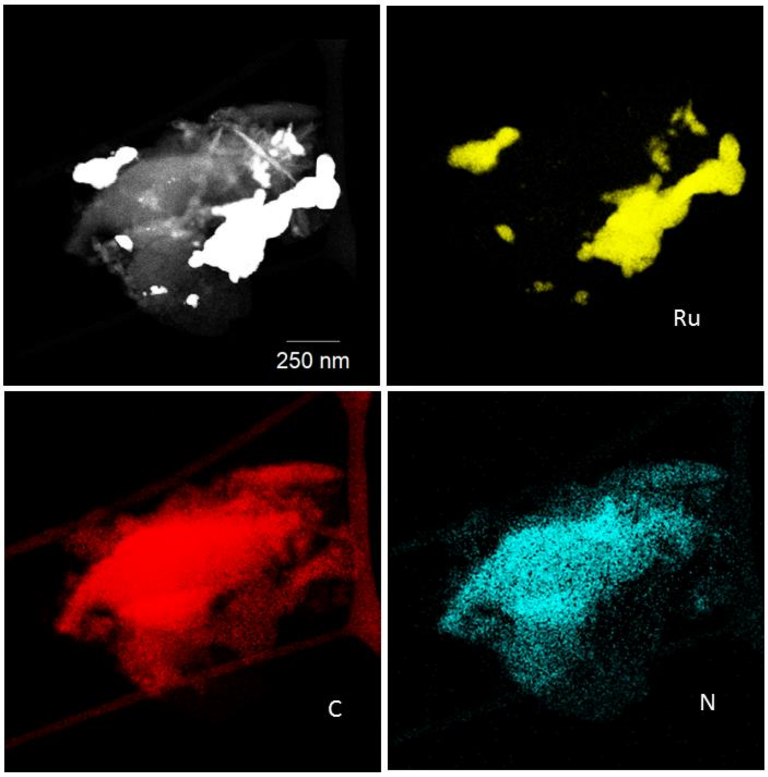

Figure S2. TEM image of Ru-CTF-900 and the corresponding EDS mapping for Ru, C and N.

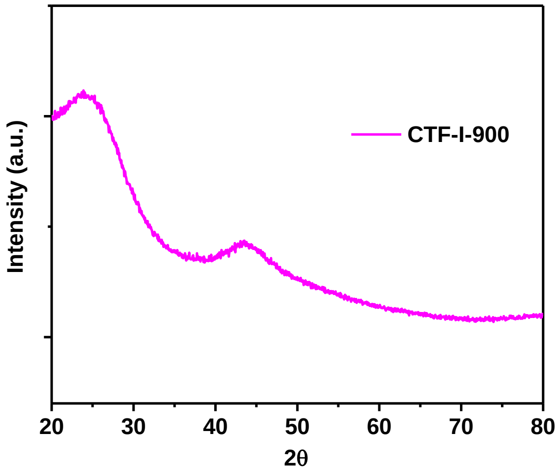

(a)

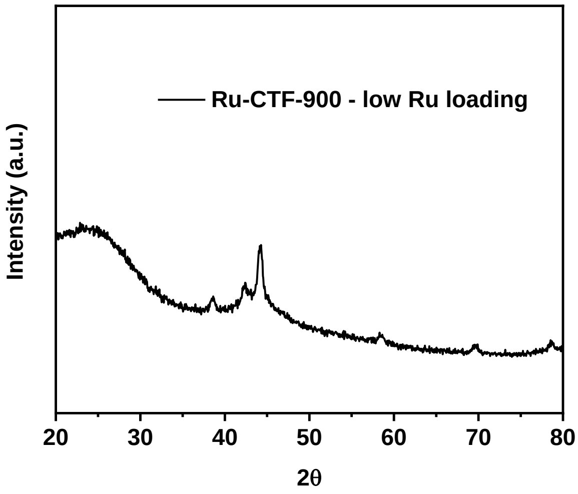

(b)

Figure S3. XRD pattern of (a) CTF-I-900 sample obtained after direct pyrolysis of CTF-I at 900 °C in N_2 atmosphere and (b) Ru-CTF-900 with lower Ru loading of ~0.6 wt%.

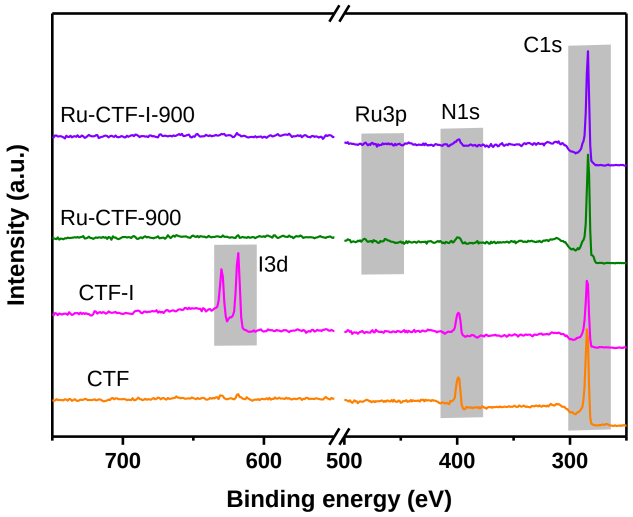

Figure S4. XPS survey spectra of CTF, CTF-I, Ru-CTF-900 and Ru-CTF-I-900 samples.

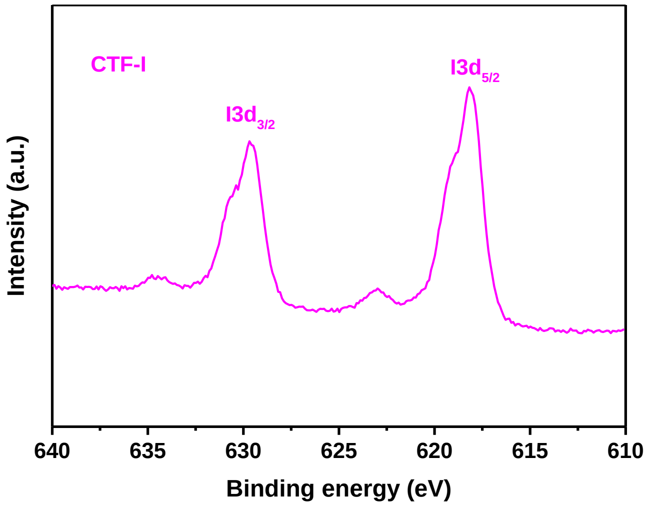

Figure S5. XPS I3d spectrum of CTF-I sample.

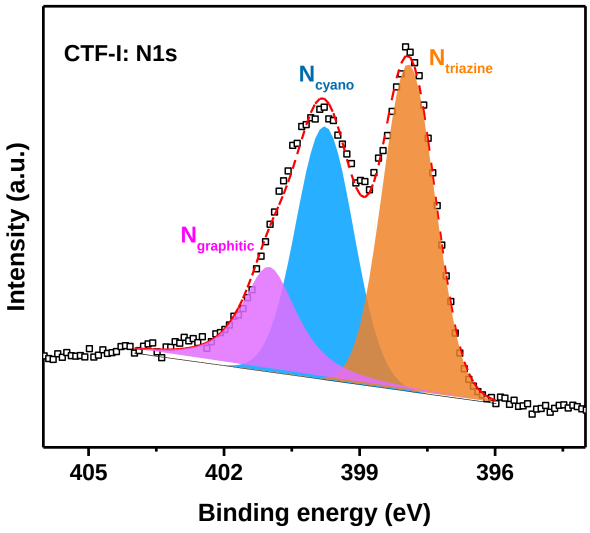

Figure S6. N1s XPS spectrum of CTF-I sample.

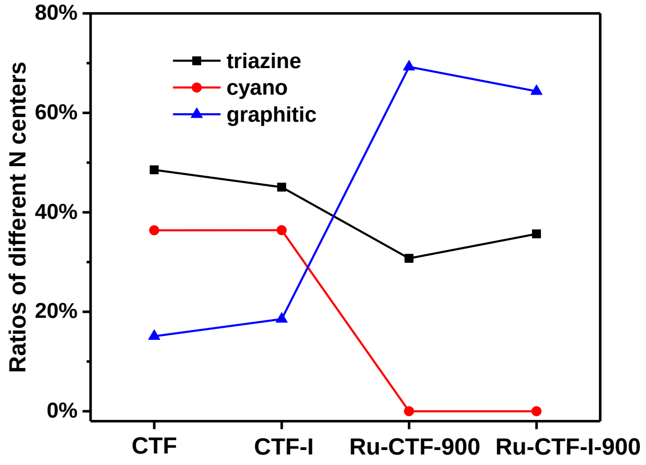

Figure S7. Ratios of $N_{triazine}$, N_{cyano} and $N_{graphitic}$ in CTF, CTF-I, Ru-CTF-900 and Ru-CTF-I-900 samples obtained from XPS results.

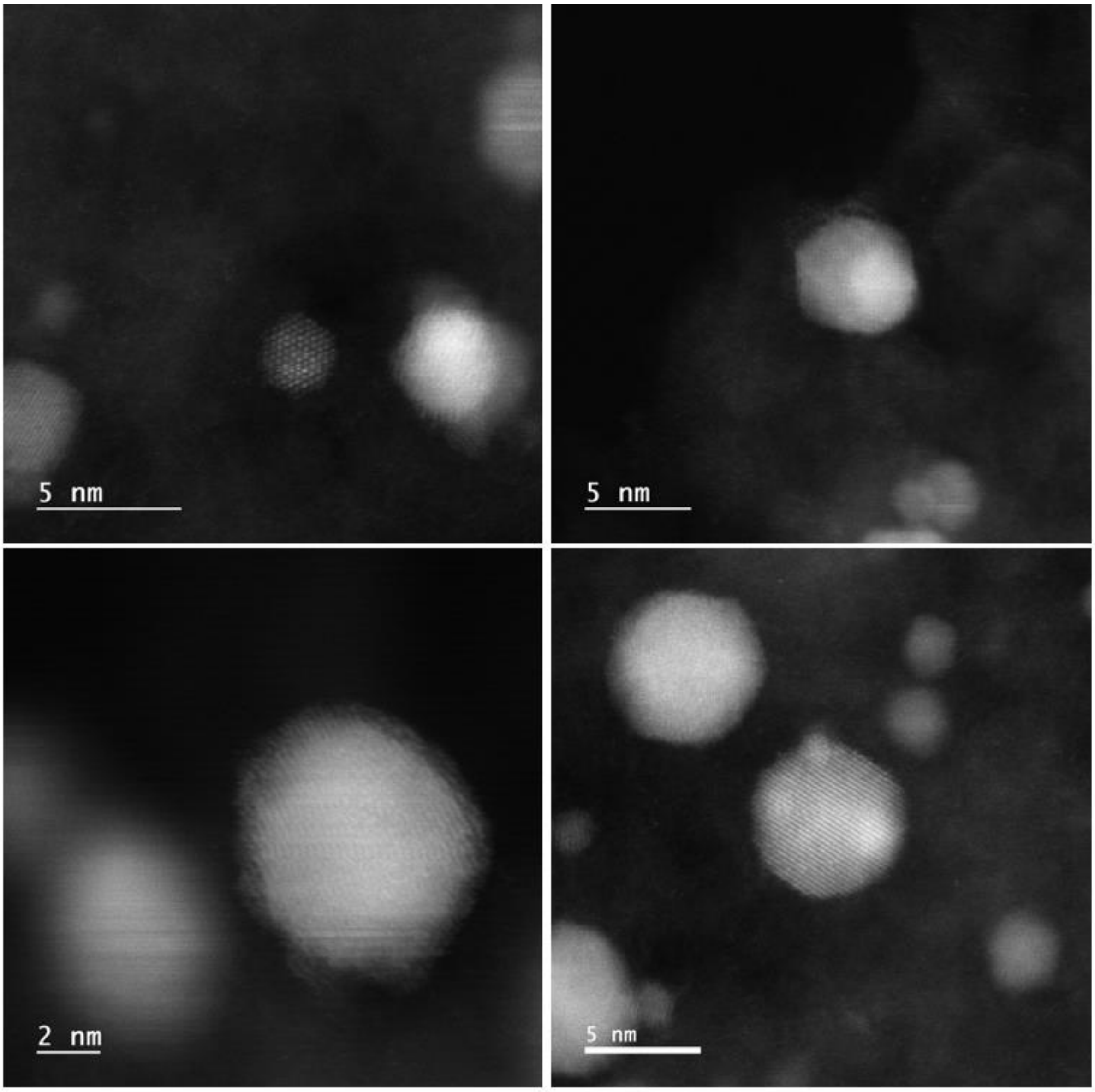

Figure S8. TEM images of Ru-CTF-I-900.

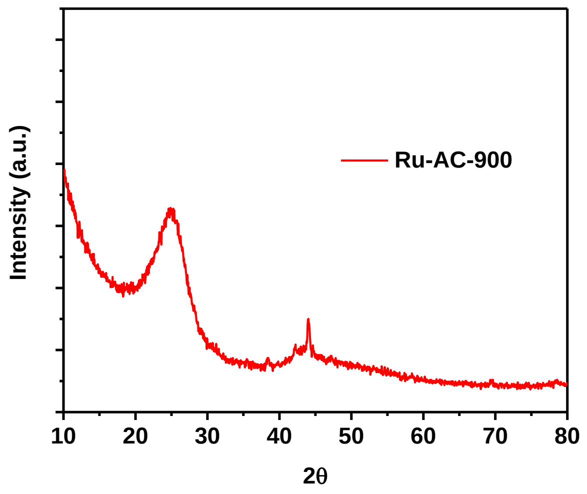

Figure S9. XRD pattern of Ru-AC-900 sample

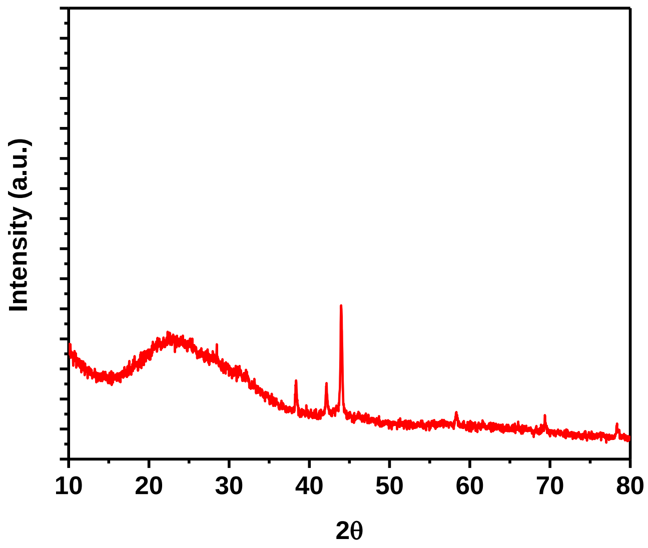

Figure S10. XRD pattern of Ru-CTF-900’ sample with $K_4[Ru(CN)_6]$ as the impregnation precursor

In order to eliminate the influence of metal precursors for Ru SACs and NPs in Ru-CTF-I-900, $K_4[Ru(CN)_6]$ instead of $RuCl_3$ was impregnated on CTF to obtain a new Ru-CTF-900 catalyst after pyrolysis at 900 °C. However, the sharp diffraction peaks ascribed to metallic Ru are observed from XRD pattern (**Figure S10**, Supporting Information), which further confirms the superiority of our charge-modulated strategy.

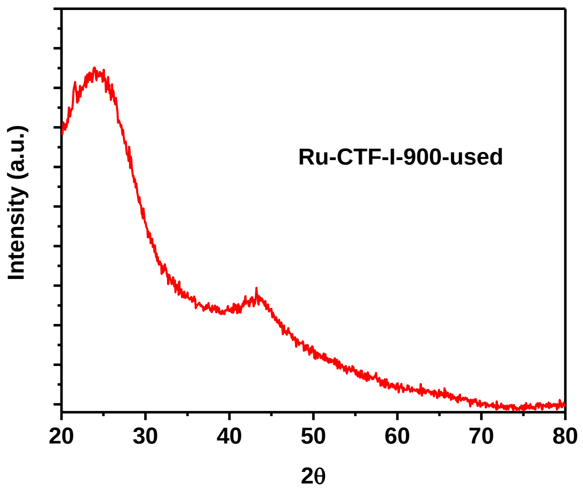

Figure S11. XRD pattern of Ru-CTF-I-900 after used for three times

[1] M.J.T. Frisch, G.W. Trucks, H.B. Schlegel, G.E. Scuseria, M.A. Robb, J.R. Cheeseman, G. Scalmani, V. Barone, B. Mennucci, G.A. Petersson, et al., Gaussian 2009, 09, Revision D. 01, Gaussian, Inc., Wallingford CT.

[2] S.F. Boys, F. Bernardi, *Mol. Phys.* 1970, 19 (4), 553-566.

[3] S. Simon, M. Duran, J.J. Dannenberg, *J. Chem. Phys.* 1996, 105 (24), 11024–11031.
